# Papillon-Lefèvre Syndrome: Diagnosis, Dental Management, and a Case Report

**DOI:** 10.1155/2019/4210347

**Published:** 2019-04-21

**Authors:** Jean-Claude Abou Chedid, Michel Salameh, Abbass El-Outa, Ziad E. F. Noujeim

**Affiliations:** ^1^Department of Pediatric Dentistry, Faculty of Dental Medicine, Saint-Joseph University, Beirut, Lebanon; ^2^Private Practice of Oral Medicine and Restorative Dentistry, Beirut, Lebanon; ^3^Department of Oral Medicine and Maxillofacial Radiology and Imaging, Faculty of Dental Medicine, Lebanese University, Beirut, Lebanon

## Abstract

**Aim:**

This paper revisits Papillon-Lefèvre syndrome (PLS), addresses its diagnostic update and dental management, and reports a case of a 5-year-old Lebanese patient with consanguineously married parents.

**Background:**

PLS, also known as “keratoris palmoplantaris with periodontopathia” and “hyperkeratosis palmoplantaris with periodontosis,” is an extremely rare autosomal-recessive trait that combines a diffuse palmoplantar hyperkeratosis and a severe generalized, progressive prepubertal form of a precocious form of juvenile, aggressive periodontitis.

**Case Description:**

We are reporting a 5-year-old boy that sustained a spontaneous loss of all his primary teeth. At consultation, he was under treatment for hyperkeratosis of his palms and soles. Detailed family history of the child revealed that the patient's parents, grandparents, and relatives were consanguineously married and two of his cousins displayed similar clinical signs (palmoplantar hyperkeratosis and premature loss of deciduous and most of the permanent teeth).

**Conclusion:**

PLS is an extremely rare disorder that usually becomes apparent from approximately 1-5 years of age. Genetic counseling should always be suggested to parents of affected children, informing them of chances of their offspring having the inherited disease.

## 1. Introduction

Papillon-Lefèvre syndrome (PLS) is a very rare genodermatosis of autosomal-recessive inheritance. It is an ectodermal dysplasia, and a type IV palmoplantar keratosis [1]. It was named, in 1924, by French physicians M.M. Papillon and Paul Lefèvre [1] and is characterized by a hyperkeratosis of soles of feet and palms of the hands (palmar-plantar) and extensive, severe, aggressive, and prepubertal periodontitis, leading to premature loss in both deciduous and permanent dentitions.

PLS prevalence is estimated between 1/250,000 and 1/1,000,000 in the general population [2, 3].

PLS disorder can be hereditary, acquired, or associated with other syndromes, but in most described cases, PLS is genetic, resulting from mutations on both alleles of the cathepsin C gene (CTSC) on chromosome 11q14.2. Most PLS patients are homozygous for CTSC mutations [4–7].

## 2. Case Report

A 5-year-old boy presented to the Department of Pediatric Dentistry of the Faculty of Dental Medicine at Saint-Joseph University of Beirut, Lebanon, with a chief complaint of loosening of all primary teeth, followed by their spontaneous loss. The patient's medical history showed that he was undergoing a dermatological treatment for the “hyperkeratosis of his palms and soles”; the treating dermatologist referred the patient to the dentist after having suspected PLS. A detailed family history revealed that the patient's parents, grandparents, and relatives were consanguineously married and two of his cousins exhibited similar clinical signs (palmoplantar hyperkeratosis and premature loss of deciduous and permanent teeth).

**Figure 1:**
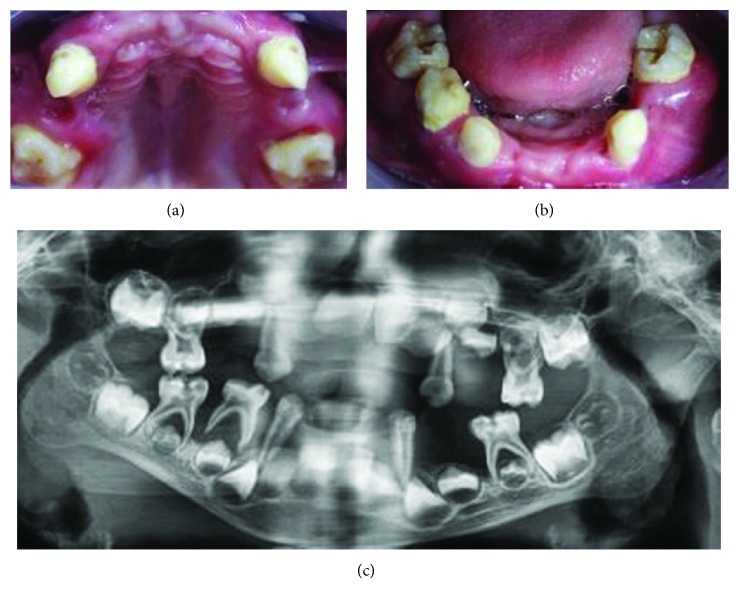
(a and b) Intraoral maxillary and mandibular aspects before multiple dental extractions. (c) Panoramic radiograph before extractions.

Intraoral examination revealed that many primary teeth (teeth 54, 52, 51, 61, 62, 64, 74, 72, 71, 81, and 82) were missing while remaining teeth (teeth 85, 84, 83, 73, 75, 65, 63, 53, and 55) exhibited plaque accumulation, multiple caries, and generalized grade III mobility with the formation of periodontal pockets (Figure 1); gingival tissues surrounding these teeth were inflamed, edematous, and tender to palpation, while those of edentulous regions appeared normal and nontender to palpation. Dental and periodontal abscesses were noticed on teeth 85 and 75. Panoramic radiograph displayed several floating teeth with generalized horizontal and vertical bone loss (Figure 1).

**Figure 2:**
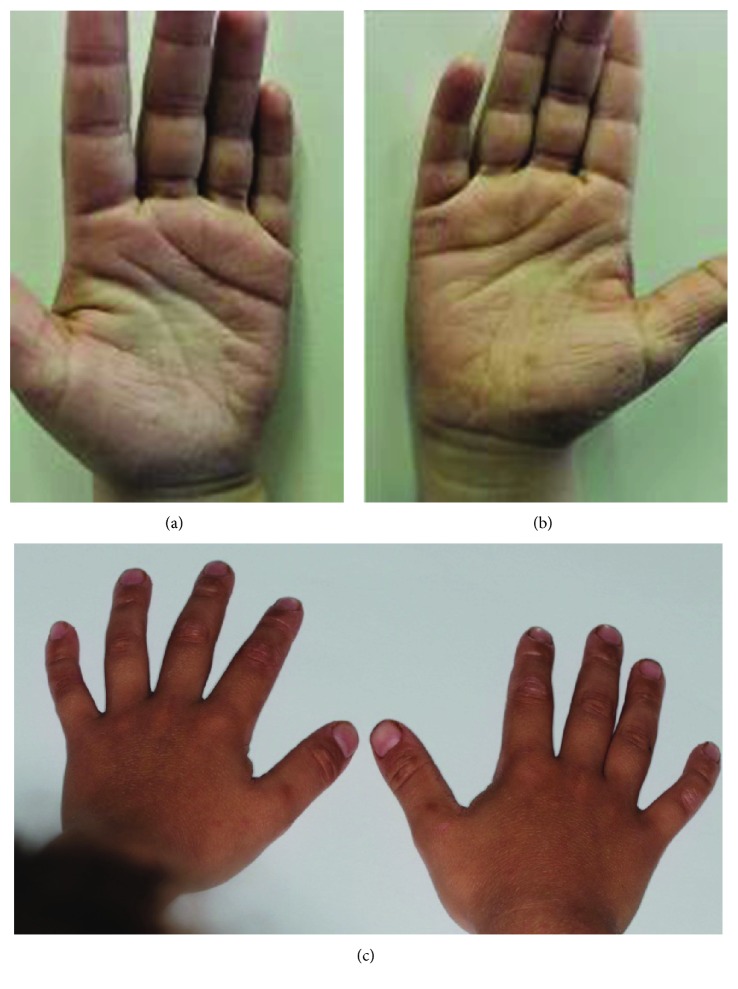
Hyperkeratosis on hand palms (a and b) and dorsal surfaces (c).

**Figure 3:**
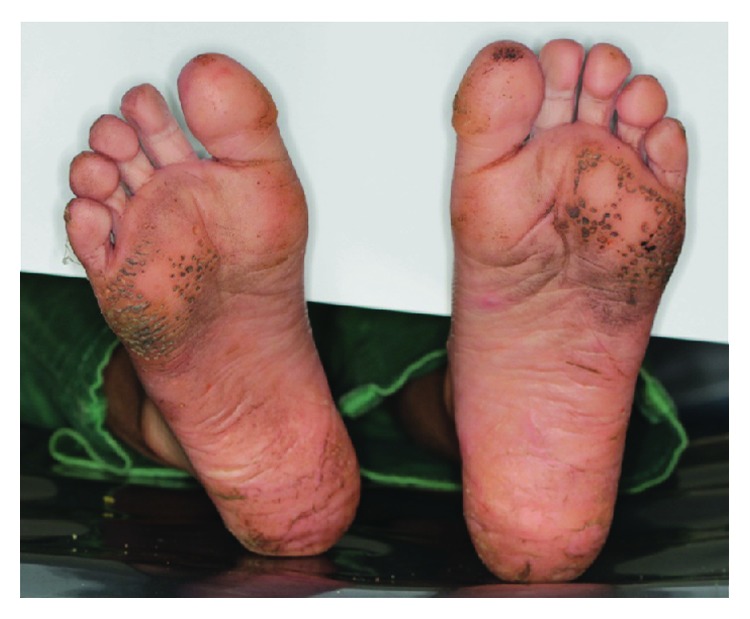
Hyperkeratosis of soles of feet (scaly and rough soles).

Extraoral examination of the patient revealed hyperkeratosis on the dorsum surface of his hands (Figure 2) and feet (Figure 3).

Routine hematological examination (CBC and blood chemistry profile) and liver function tests were normal. In addition, a genetic test was performed at the Saint Joseph University Faculty of Medicine/Laboratory of Molecular Biology, Beirut; PCRs (polymerase chain reaction) followed by fluorescent Sanger sequencing of exons 3 to 7 of *CTSC* gene, for the affected child, revealed no mutation. However, PCR amplification of exons 1 and 2 did not result in any product, suggesting that affected child harbored a homozygous deletion of the 5′UTR region and exons 1 and 2.

Level of patient's cooperation was assessed during the first clinical examination, and it was decided to implement the treatment under general anesthesia, at hospital. A written informed consent form was signed by the child's parents after having discussed with them all the steps of the treatment planning, from extraction of all remaining teeth to the fabrication of an immediate removable complete denture (RCD). The importance of oral hygiene was emphasized, and enforcement of oral hygiene habits was advocated.

At hospital, the first dose of amoxicillin (50 mg/kg) and clavulanic acid was administered through IV route before beginning the surgery and it was continued for the following postoperative seven days. In order to maintain a stable occlusion, two impressions of maxilla and mandible were taken with the remaining teeth in place using an addition silicon material. All remaining teeth were extracted, and all extraction sockets were irrigated with a 0.12% chlorhexidine digluconate solution. No sutures were needed, and impressions were directly transferred to the dental laboratory in order to be casted.

**Figure 4:**
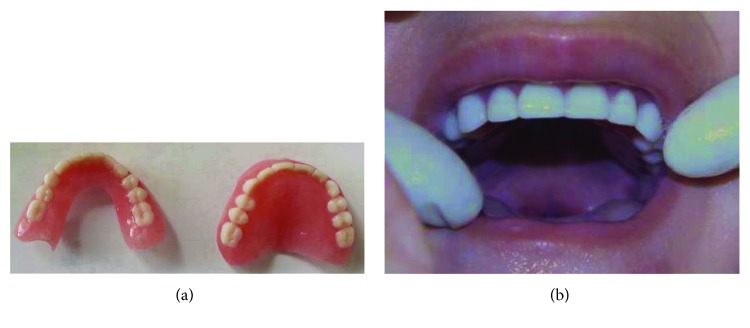
(a) Maxillary and mandibular removable complete dentures. (b) Insertion of maxillary denture in the mouth.

**Figure 5:**
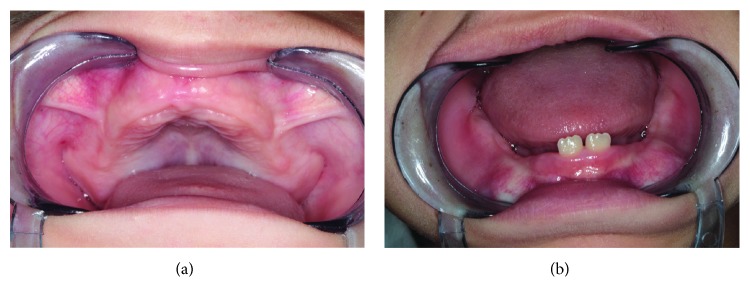
The erupting mandibular central incisors.

Two weeks after extractions, maxillary and mandibular RCDs were fabricated (Figure 4(a)) and inserted in the mouth (Figure 4(b)). No pressure spots were noticed, and occlusion showed no interferences. Patient and parents were taught the proper oral hygiene measures for RCDs; they were also informed of the need for regular dental visits (one week after the insertion and every three months afterwards) in order to reexamine the appliances and, when necessary, replace or reline them according to arch growth and eruption of the permanent teeth. Eight-month follow-up revealed the erupting mandibular central incisors (Figure 5).

## 3. Discussion

### 3.1. Consanguinity in PLS

Consanguineous marriage [8] is matrimony between persons who are closely related. It is defined as a union between two individuals who are related as second cousins or closer, with an inbreeding coefficient (*F*) equal or higher than 0.0156; *F* representing a measure of the proportion of loci at which the offspring of a consanguineous union is expected to inherit identical gene copies from the father and mother. This includes unions termed first cousins and second cousins. In some Arab communities where unions occur between double first cousins, the highest inbreeding coefficients are reached, and in some regions of South India where uncle-niece marriages occur, *F* reaches 0.125 [8].

Increased genetic risks to the offspring represent the main negative impact of consanguineous marriages. Indeed, consanguineous unions may lead to an increased expression of autosomal-recessive disorders, and the closer the biological relationship between parents, the greater is the probability that their offspring will inherit similar copies of one or more detrimental recessive genes [9]. Consanguinity does not increase the risk for autosomal-dominant diseases when only one of the parents is affected nor for X-linked recessive conditions if neither parent is affected [8].

Premarital screening and preconception counseling programs are important in raising public's awareness of possible genetic diseases and their prevention by knowing more on consanguinity as a risk factor for the expression of recessive disorders. In Shiraz, Southern Iran, among 2,686 couples presenting for genetic counseling during a period of 4 years, 85% had consanguineous relationships and 74% were first cousins [10].

In preconception counseling for consanguinity, it is important to make the difference between families with no known genetic disorder and those with a known inherited or genetic one. Taking a detailed family history and constructing a four-generation pedigree (offspring, siblings, parents, aunts, uncles, grandparents, nieces, nephews, and first cousins) are obligatory [9]. Collection of information is implemented by addressing very specific questions that could lead to the detection of a genetic or hereditary disorder in the extended family; these questions should include any relevant information about any of the following pathologies in blood relatives:

1. Congenital anomalies or birth defects
2. Learning disability or mental retardation (or disability)
3. Inherited blood disorder
4. Undiagnosed severe condition or anomaly
5. Early vision and/or hearing impairment
6. Failure to thrive or developmental delay
7. Epilepsy
8. Unexplained infant (or neonatal) death in the offspring

Consanguinity is a deeply rooted social trend among one-fifth of the world population mostly residing in the Middle East, North Africa, and West Asia, as well among some emigrants from these regions now residing in Europe, North America, and Australia. In these highly consanguineous populations and communities, premarital counseling on consanguinity is almost absent. Around one billion of the current global population live in regions and communities with an obvious preference for consanguineous (blood relatives) marriages. In such communities and regions, though cousin marriages are customary, there are inconsistencies among healthcare providers in counseling for consanguinity possible consequences and sequelea [9].

When PLS is genetic, it is transmitted as an autosomal-recessive trait, and 20-40% of patients are children of consanguineously married parents [11].

In their original paper, back in 1924, Papillon and Lefèvre reported [1] the case of two siblings who were the products of a first-cousin mating, and the condition described in their paper featured two hallmarks which were a premature loss of deciduous and permanent dentitions and diffuse transgradient palmoplantar keratosis. Consanguinity is now recognized as a risk factor [12] for PLS, and many articles reported PLS cases in the same family [13–15]. Considering that consanguineous marriage is customary in Lebanese society, potential parents are to be warned that this leads to an increased birth prevalence of infant with severe recessive disorders; consequently, this kind of marriage should be discouraged, and genetic counseling should be advocated. Families at increased risk should be identified, and carrier testing should be implemented when feasible [16].

### 3.2. Clinical Manifestations

PLS becomes clinically apparent from the ages of 1 to 5; dry scaly patches of palms and soles (palmar-plantar hyperkeratosis) and severe, aggressive, and early-onset periodontitis are its main clinical features, but additional symptoms may be associated to it, such as pyogenic skin infections, nail dystrophies, and hyperhidrosis. Patches of the skin usually develop around the age of 1 to 5; they are mostly confined to the undersides of feet and hands, but they may appear on elbows and knees. In rare cases, upper portions of hands and feet, eyelids, and other parts of the body may be affected as well. Affected skin is often thick and red, but texture and color may vary; skin lesions may worsen during cold weather, and walking is often uncomfortable and painful.

Patients affected with PLS may also exhibit generalized hyperhidrosis (excessive perspiration), leading to unpleasant, malodorous odors. Most patients with PLS have hypotrichosis (sparse hair on the scalp and body), dirty colored skin, and very fragile nails that easily break off, but some of them may release excessive amounts of keratine in hair follicles, causing rough papules on the skin.

Only some individuals affected by PLS may experience other kinds of infections such as furunculosis, respiratory tract infections, and hidradenitis suppurativa (painful and swollen lesions in the breast, groin, and armpit).

### 3.3. Craniofacial, Oral, Dental, and Periodontal Aspects

Dental and periodontal manifestations [3] of PSL become apparent by the age of 2-3 years; they start with severe gingivitis and rapid periodontal destruction, followed by premature exfoliation of all deciduous teeth by the age of 4 to 5. The same sequence of events recurs as the permanent dentition erupts, leading to its early shedding, and without treatment, most of the permanent teeth may fall by the same mechanism by the age of 16 years. Consequences of this premature tooth loss will affect the patient's daily functions, such as mastication and speech.

Gingival and periodontal tissues are involved (gingivitis, gingival ulcers, periodontal pockets, and severe, aggressive periodontitis) in PLS. Aggregatibacter actinomycetemcomitans plays an important role in the progression of periodontal infections and inflammations. Treponema denticola, Porphyromonas gingivalis, and Fusobacterium nucleatum are also suggested as causative agents [17].

At eruption of deciduous teeth, gums appear red, inflamed, and sometimes bleedy and enlarged. In some cases, all oral mucosae may appear erythematous (stomatitis), with possible perioral lymphadenopathies. Halitosis may also develop in some individuals. Apart from that, discrepancies in the arch length and psychological problems may follow [18].

In rare people affected with PLS, dry scaly patches of the skin (hyperkeratosis) may appear on lips, cheeks, and eyelids, and in some rare adult patients, cysts may develop on eyelids and calcium may accumulate in the skull, causing intracranial calcifications [19, 20].

### 3.4. Differential Diagnosis

To distinguish between PLS and palmoplantar hyperkeratosis syndromes, occurrence of severe, aggressive, and early-onset destructive periodontitis has to be taken into consideration [21].

Many disorders share similar clinical and genetic features with PLS [22]:

1. The association with severe, aggressive, and early-onset periodontitis is unique to PLS and Haim-Munk syndrome (HMS). HMS (known also as “palmoplantar keratoderma with periodontitis and arachnodactyly and acroosteolysis” or “Cochin Jewish disorder”) is a skin condition caused by a cathepsin C gene mutation; it is an extremely rare disorder of keratinization of recessive inheritance that manifests with scaly, red, and thickened patches of the skin of soles of the feet and palms of the hands (palmoplantar hyperkeratosis), pes planus (flat foot), arachnodactyly (a peculiar deformity of terminal phalanges of hands and feet), acroosteolysis, atrophic changes of nails (onychogryphosis), a radiographic deformity of fingers, recurrent abscess formations, and a severe early-onset periodontitis. PLS cases were reported worldwide while HMS ones were only described among descendants of a religious isolate originally from Cochin, India. Consanguinity is a major feature of both conditions.
2. Palmoplantar ectodermal dysplasia (PPED) is a skin condition that includes 8 types: PPED type 1 or pachyonychia congenita which is autosomal dominant, PPED type 2, PPED type 3 or acrokeratoelastoidosis, PPED type 4 which resembles the PLS, PPED type 5 or oculocutaneous tyrosinemia type II or Richner-Hanhart syndrome which is autosomal recessive, PPED type 6 or Olmsted syndrome, PPED type 7, and PPED type 8 or Meleda disease or “mal de Meleda” or “acral keratoderma” which is autosomal recessive.
3. von Feer's disease (acrodynia, pink disease, mercurialism, erythredema, and Swift-Feer disease) affects children which is characterized by dusky pink discoloration in hands and feet, loss of teeth, hair, and nails, red lips, cheeks, and nose, transient rashes, hypotonia, photophobia, kidney dysfunction, peripheral neuropathy (paresthesia, itching, burning sensation, and pain), and neuropsychiatric symptoms (insomnia, emotional lability, and memory impairment). Mercury intoxication/poisoning is incriminated as a causative factor of this disease.

Other pathologies and syndromes sharing clinical features with PLS [22] include palmoplantar hyperkeratosis (Unna-Thost syndrome), palmoplantar hyperkeratosis-punctate, Vohwinkel's syndrome, Vörner's localized epidermolytic palmoplantar keratoderma, Howel-Evans syndrome, Gamborg-Nielsen syndrome, and Greither's transgrediens and progrediens palmoplantar keratoderma.

Some PLS patients were reported to have palmoplantar keratosis and periodontal destruction in their permanent dentition, with no periodontal destruction observed in their deciduous one [21, 23, 24].

A cohort study including 47 patients [25] supported the concept that the two major components of PLS (periodontal destruction and palmoplantar keratosis) appeared to be unrelated to each other; in this study, skin and oral changes developed early in life, with no exception. Dermatological involvement showed no correlation with age, while periodontal infection was significantly worse in young age with primary teeth. Finally, a strong correlation was found between feet and hand condition, although the scores for the feet were significantly higher, and no significant correlation could be demonstrated between the severity of skin affections and the level of periodontal destruction [25].

## 4. Conclusion

PLS periodontosis ultimately leads to premature loss of deciduous and permanent dentitions at a very young age. This devastating periodontitis is attributed to a point mutation of the cathepsin C gene. Prognosis of dentition in these patients is poor, and most of them ultimately become edentulous, except for third molars, and end up with a small maxilla and mandible. In almost half of the patients, PLS displays an enhanced susceptibility to systemic and cutaneous infections, such as skin abscesses, furunculosis, hidradenitis suppurativa, and respiratory tract infections. Patients may also sustain nail dystrophies, follicular hyperkeratosis, dural calcifications, and malodorous hyperhidrosis; on very rare occasions, PLS may be associated with squamous cell carcinoma and malignant melanoma.

Conventional treatment of PLS is mainly based on the use of oral retinoids which often attenuate signs of palmoplantar keratoderma and, somehow, slow the resorption of the alveolar bone on the remaining teeth. Etretinate, a synthetic retinoid, lately showed very promising results in the medical treatment of PLS. Antibiotics are also prescribed in treating recurrent infections.

A very good oral hygiene with concomitant use of chlorhexidine mouth rinses should also be recommended to patients in order to slow the aggressive progression of periodontitis. Finally, deciduous and remaining teeth are often extracted and replaced, either by conventional removable dentures or dental osseointegratable implants.

Successful periodontal management of PLS patients is the key to improve the prognosis of the dentition, preventing or delaying primary and permanent tooth loss. It is mandatory that all dental professionals be familiar and aware of this disease as well as the importance of its early diagnosis and management.
